# Systematic review: interventions to quit tobacco products for young adults

**DOI:** 10.1186/s12889-023-15900-8

**Published:** 2023-06-26

**Authors:** Eunhee Park, Yanjun Zhou, Chiahui Chen, Thomas Chacko, Martin Mahoney, Yu-Ping Chang

**Affiliations:** 1 University at Buffalo, School of Nursing, 3435 Main St, 14214-8013 Buffalo, NY US; 2 Roswell Park Comprehensive Cancer Center, Elm and Carlton Streets, Buffalo, NY 14263 US

**Keywords:** Smoking cessation, Young adults, Systematic review

## Abstract

**Background:**

Young adulthood is an important period for smoking cessation; however, there is limited evidence of smoking-cessation interventions for young adults. The aims of this study were to identify evidence-based smoking-cessation strategies for young adults, examine gaps in the literature regarding smoking cessation among young adults, and discuss methodological issues/challenges related to smoking-cessation studies for young adults.

**Methods:**

Studies tested interventions for smoking cessation among young adults (18 to 26 years old), excluding pilot studies. Five main search engines were used, including PubMed, the Cumulative Index of Nursing and Allied Health Literature (CINAHL), EMBASE, PsycINFO, and Web of Science. The search was conducted for articles published from January 2009 to December 2019. Intervention characteristics and cessation outcomes were reviewed, and methodological quality was evaluated.

**Results:**

A total of 14 articles met inclusion criteria, including randomized controlled studies and repeated cross-sectional studies. Interventions included the following: text messaging (4/14, 28.6%), social media use (2/14, 14.3%), web-or app-based intervention (2/14, 14.3%), telephone counseling (1/14, 7.1%), in-person counseling (3/14, 21.4%), pharmacological (1/14, 7.1%), and self-help booklet (1/14, 7.1%). The intervention duration and frequency of contact with participants differed and yielded varied outcomes.

**Conclusions:**

Multiple interventions have been examined to aid young adults in achieving smoking cessation. While several approaches seem promising, at the present time, the published literature is inconclusive about the type of intervention that is most effective for young adults. Future studies should compare the relative effectiveness of these intervention modalities.

**Supplementary Information:**

The online version contains supplementary material available at 10.1186/s12889-023-15900-8.

## Background

Although smoking prevalence has been declining overall, persons aged 18–24 currently show high smoking rates (17.1% [1]), and 99% of smokers become regular users by age 26 [2]. Quitting before 30 years of age can significantly reduce tobacco-related morbidity and mortality [3], as half of smokers will eventually die because of smoking-related disease [4]. Although young adults try to quit smoking, they often fail to quit [5]. Furthermore, young adults are less likely to receive help from health care providers during their attempts to stop smoking [6, 7].

In contrast to a broad base of cessation studies among mid-adult and older smokers, here have been only a limited number of smoking-cessation intervention studies targeted to young adults. Although various definitions exist, young adults were defined as persons age 18 to 26 years who share unique developmental characteristics and confront important social challenges in their lives [8, 9]. Previous systematic reviews have identified various gaps in smoking-cessation interventions for young adults [10, 11]. For instance, the Cochrane review analyzed randomized controlled studies of cessation focused on persons under 20 years [10]. Overall, Fanshawe and colleagues found that 1) group-based interventions were positive, 2) interventions using stage of change models, motivational interviewing (MI), and social cognitive effect resulted in small effect sizes, and 3) interventions based on complex models showed the most promising results. In addition, only a small number of studies used pharmacological interventions, which showed very small effect sizes and were generally underpowered. However, this review did not analyze data based on age and reported the outcomes separately for young adults. Another review focused on smoking-cessation interventions for young adults (18 to 24 years) published between 2009 and 2019 [11], and reported promising results for interventions based on the use of socio-cognitive theory, quit line counseling, and text messaging. Although that review provided important insights regarding smoking cessation interventions for young adults, it did not include pharmacologic interventions or interventions focusing on use of tobacco products other than cigarettes. Given that e-cigarettes are the most prevalent and emerging tobacco products in this population, there is a critical need to examine interventions for diverse tobacco products, not only cigarette smoking cessation.

### Purpose of this study

Despite the extant literature, there are gaps remain in terms of which approaches to young adults’ smoking-cessation interventions are most effective. Thus, the purpose of this systematic review was to examine the interventions to quit young adults’ tobacco products. The aims of this study were to (1) identify evidence-based strategies to quit tobacco products for young adults, (2) examine gaps in the literature regarding quitting tobacco products among young adults, and (3) discuss methodological issues/challenges related to the intervention studies for young adults to quit tobacco products.

## Methods

### Study protocol

This systematic review followed the Preferred Reporting Items for Systematic Reviews and Meta-Analysis guidelines [12].

### Search strategies

**Fig. 1:**
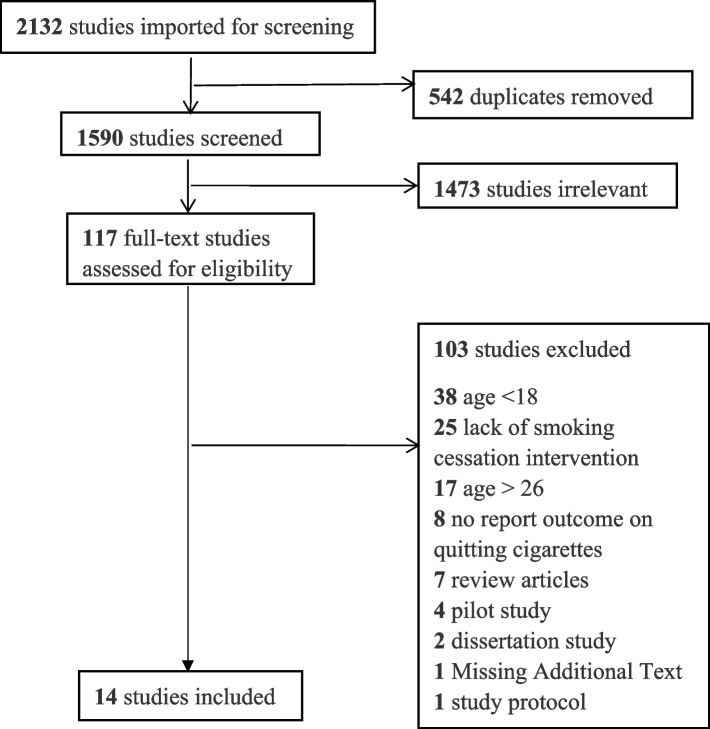
PRISMA Process of Reviewing Articles

Five search databases were used to identify studies: PubMed, CINAHL, EMBASE, PsycINFO, and Web of Science. Figure 1 provides detailed information on the search results. Search terms were combined from three categories, including the MeSH and non-MeSH terms (1) young adults (“young people” or “youth” or “adolescents” or “emerging adults”, or “young adults” [MeSH]); (2) “smoking” or “tobacco” or “cigarette” or “nicotine”, or “cigarette smoking [MeSH], or “Tobacco [MeSH], “Tobacco use [MeSH], “smoking [MeSH], OR “e-cigarettes” OR “electronic cigarettes” OR “vapor cigarettes” OR “vapes” OR “electronic nicotine delivery device” OR “juul” OR “electronic vaporizer” OR “electronic nicotine” OR “electronic nicotine delivery systems [MeSH]” OR “vaping [MeSH]”; and (3) intervention (“intervention” or “treatment” or “therapy” or “cessation” or “prevention”, “stopping” or “quitting” or “smoking cessation” or “tobacco use cessation” or “smoking stopping” or “quitting smoking” or “giving up smoking”, “therapeutics” [MeSH], or “smoking prevention” [MeSH]) by using “AND” as the conjunction word for three collections of terms. Studies were included if they were peer-reviewed articles published in academic journals between January 2010 and December 2019. The search process was conducted from January 2020 to September 2021.

### Inclusion criteria

According to the predetermined inclusion criteria, studies were included if (1) they were published in a peer-reviewed journal between 2010 and 2019; (2) they tested smoking-cessation interventions and reported outcome-related cessation of tobacco products, including combustible and non-combustible products using randomized controlled study design; (3) the study population consisted of young adults aged 18 to 26 years; and (4) full articles were available in English. Articles were excluded if (1) they were not intervention studies, (2) they did not provide smoking-cessation-related outcomes, or only provided the feasibility or protocol of the intervention or they were pilot studies; gray literature including dissertations, conference proceeding papers, abstracts, or editorials were also excluded.

### Selection process

After searching from the search engines, Covidence program was used to manage the selection process [13]. Two reviewers independently reviewed each article to check the eligibility. When a discrepancy was found, a 3^rd^ reviewer was included in the discussion to reach a final decision.

### Data extraction, analysis, and synthesis

The final 14 articles were fully reviewed. The first step was to perform a descriptive evaluation of each study by presenting them in a tabular format. Based on the purpose of this review, the following information from the articles was extracted into an Excel file: authors, year of publication, country, city, setting, sample size, age of participants (range and/or mean age), female proportion, study design, intervention characteristics (mode, frequency, duration, and intensity), control group condition, and quit rates. One researcher conducted the initial coding for the raw-data coding process. Next, two other researchers separately checked the accuracy of the coded information against the original articles by carefully reading the full articles. The information was reorganized and formatted as Tables 1, 2, and Supplement Table 1. Next, the authors grouped studies based on the types of intervention approaches and reported the study outcomes based on whether they produced positive quitting outcomes (e.g. higher quit rates and statistical significance) compared to the control groups. The findings are descriptively presented in the Table 2. Descriptive statistics was used. In addition, we examined gaps in the literature regarding quitting tobacco products among young adults (e.g. subpopulation, tobacco products, and approaches). Finally, we examined methodological issues and challenges of the identified intervention studies for quality evaluation using the quality evaluation guidelines from the Agency for Healthcare Research and Quality (AHRQ) [14]. Evaluation criteria were used for 10 domain areas: study question, study population, comparability of subjects, randomization, blinding, interventions, outcomes, statistical analysis, results, and discussion. Table 3 describes the detailed criteria, and two authors reviewed each article. If discrepancies occurred, external review was considered to solve the inconsistencies.

**Table 1:** Samples and Settings of the Intervention Studies

	Authors	Tobacco products	Country	City, Setting	N	Age (in years, mean or range; Female proportion)
Text-based	Haug et al., 2013 [15]	cigarettes	Switzerland	Vocational schools	755	Mean = 18.3 (51.9%)
	Skov-Ettrup et al., 2014 [16] (Tailored text messages)	cigarettes	Denmark	Online	2030	15–25 (59.3%)
	Ybarra et al., 2013 [17] (SMS USA)	cigarettes	USA	Online	164	18–25 (44%)
	Graham et al., 2021 [18] (SMS USA)	vape	USA	Online	2,588	18–24 (50.3%)
Social media	Ramo et al., 2018 [19] (Tobacco Status Project Facebook)	cigarettes	USA	Online	500	18–25 (55%)
	Vogel et al., 2019 [20]	cigarettes	USA	Online	500	18–25 (54.6%)
App	Baskerville et al., 2018 [21]	cigarettes	Canada	Online	1599	19–23 (45.94%)
Web-based & App	Epton et al., 2014 [22]	cigarettes	UK	University of Sheffield	1445	Mean = 18.9 (58%)
Phone or virtual counseling	Sims et al., 2013 [23] (Quit line)	cigarettes	USA	Seattle	410	18–24 (59.2%)
In-person counseling	Zanis et al., 2011 [24] (Brief direct treatment intervention)	cigarettes	USA	6 rural Pennsylvania counties	192	18–24 non-college students (57.5%)
	Harris et al., 2010 [25]	cigarettes	USA	One large Midwestern university	452	Mean = 19.45 (46.5%)
	Orsal & Ergun, 2021 [26]	cigarettes	Turkey	Youth Friendly Center (YFC) Istanbul	1854	18–20 (0.06%)
Booklet-based + a booster phone call	Travis & Lawrance, 2009 [27] (Smoke Quit)	cigarettes	Canada	6 public universities in Ontario	395	Mean = 21.37 (50.5%)
Pharmacological intervention	Tuisku et al., 2016 [28] (Nicotine patch)	cigarettes	Finland	Hospital in Rovaniemi	291	18–26 (50.2%)

**Table 2:** Outcomes of the Intervention Studies

Intervention	Studies (Intervention)	Control group	Study Design	Quit Rates and Study Outcomes	
Text-based	Haug et al., 2013 [15]	Assessment only control group	RCT	[SR]^c^ 7-day abstinence rates: 12.5% (control group: 9.6%, *p* > .05) at 6 month f/u 30-day abstinence rates: 6.3% > (control group: 5.5%), *p* > 0.05) at 6 month f/u	-
	Skov-Ettrup et al., 2014 [16] (Tailored text messages)	Untailored text messages	RCT	[SR] 30-day abstinence rates: 18.8% (control group: 13.8%, *p* > .05) at 12 month f/u	-
	Ybarra et al., 2013 [17] (SMS USA)	Attention-matched control group (sleep and physical activity)	RCT	[SR] 7-day abstinence rates: 44% (control group: 27%, *p* > .05) at 1 month f/u 3-month abstinence rates: 40% (control group: 30%, *p* > .05) at 3 month f/u	-
	Graham et al., 2021 [18] (SMS USA)	Assessment only control group	RCT	[BV&SR]30-day abstinence rates: 24.1% (control group: 18.6%) at 7 month f/u (*p* < .05)	+
Social media	Ramo et al., 2018 [19] (Tobacco Status Project Facebook)	Referral to Smokefree.gov	RCT	[BV]^c^ 7-day abstinence rates: 8.3% (control group: 3.2%, *p* > .05) at 3 month f/u 7-day abstinence rates: 6.2% (control group: 6.0%, *p* > .05) at 6 month f/u 7-day abstinence rates: 5.9% (control group: 10.0%, *p* > .05) at 1 year f/u [SR] 7-day abstinence rates: 13.6% (control group: 7.5%, *p* > .05) at 3 month f/u 7-day abstinence rates: 18.6% (control group: 14.5%, *p* > .05) at 6 month f/u 7-day abstinence rates: 21.8% (control group: 20.8%, *p* > .05) at 1 year f/u	-
	Vogel et al., 2019 [20]	Referral to Smokefree.gov	RCT	[SR] 7-day abstinence rates: 8.6% (SGMs)^d^ and 15.4% (non-SGMs) 3 month f/u (*p* > .05) 7-day abstinence rates: 18.8% (SGMs) and 15.4% non-SGMs at 6 month f/u (*p* > .05)	-
Mobile App	Baskerville et al., 2018 [21]	Self—help booklet	RCT	[SR] Intervention: 7.3%Control group: at 6.1% (50/820) for 7.3%—for OnRQ at 6 month f/u (*p* > .05)	-
Web-Based & Mobile app	Epton et al., 2014 [22]	Assessment only control group	RCT	[BV] 6-month f/u had less smokers in the intervention group (8.7%) versus control group (13.0) (*p* < .05)	+
Phone or virtual counseling	Sims et al., 2013 [23] (Quit line)	Self-help booklet	RCT	[BV] 7-days abstinence rate rates: 11% (Control group: 8.5%, *p* > 0.05) at 1 month f/u 8.1% (Control group: 7.5%, *p* > 0.05) at 3 month f/u 6.7% (Control group: 6.5%, *p* > 0.05) at 6 month f/u	-
In-person counseling	Zanis et al., 2011 [24] (Brief direct treatment intervention)	Telephone quitline (TQ)	RCT	[BV] 30-day abstinence rates: 19.8% (TQ group: 10.2%, *p* > 0.05) at 3 month f/u	-
	Harris et al., 2010 [25]	Attention-matched control group (fruits and vegetables)	RCT	[BV] 30-days abstinence rate rates: 20.4% (Control group: 24.6%, *p* > 0.05) at 6 month f/u [SR] 30-days abstinence rate rates: 31.4% (Control group: 28%, *p* > 0.05) at the post-intervention	-
	Orsal & Ergun, 2021 27]	Peer education	RCT	[SR] The score of the Fagerstrom nicotine addiction test decreased from 10 to 0.5 in control group and from 9.7 to 0.2 in intervention group (*p* < 0.001)	+
Booklet-based + a booster phone call	Travis & Lawrance, 2009 [27] (Smoke Quit)	(1) Cancer Society’ program (One-step at a Time) (2) Usual care	RCT	[SR] 7-day abstinence rates: 11.4% (Control groups: (1) 2.9%, (2) 5.6%, *p* < 0.05) at 3 month f/u	+
Pharmacological intervention	Tuisku et al., 2016 [28] (Nicotine patch, Varenicline)	Placebo treatment	RCT	[SR] 7-day abstinence rates of Nicotine patch 10ming/16 h: 26.6% (placebo control group: 19.8%, *p* > .05) at 1 month f/u 23.4% (placebo control group: 17.4%, *p* > 0.05) at 3 month f/u 20.2% (placebo control group: 15.1%, *p* > 0.05) at week 26 (*p* > 0.05) [SR] 7-day abstinence rates of Nicotine patch 15 mg/16 h: 19.6% (Varenicline group: 73.3%, *p* < .05) at 1 month f/u 15.7% (Varenicline group: 36.7%, *p* < 0.05) at 3 month f/u 9.8% (Varenicline group: 18.3%, *p* > 0.05) at week 26 (*p* > 0.05)	±

a. Results in the control group were not provided

b. N/R: Not reported

c: SR: self-reported; BV: biochemically verified

d. SGM: sexual and gender minority

e. + : statistically significant

f. -: statistically non-significant

g. ± : mixed results (statistically significant and non-significant)

**Table 3:** Evaluation of Study Quality

First Author, year	Study question^a^	Study population^b^	Comparability of subjects^c^	Randomization^d^	Blinding^e^	Interventions^f^	Outcomes^g^	Statistical analysis^h^	Results^i^	Discussion^j^
Baskerville et al., 2018 [21]	●	●	N/A	◐	●	●	●	●	●	●
Epton et al., 2014 [22]	●	●	N/A	●	○	●	●	●	●	●
Graham et al., 2021 [18]	●	●	N/A	●	●	●	●	●	●	●
Harris et al., 2010 [25]	●	●	●	◐	○	●	●	●	●	●
Haug et al., 2013 [15]	●	●	●	●	●	●	●	●	●	●
Orsal & Ergun, 2021 [26]	●	●	●	●	○	●	●	◐	●	●
Ramo et al., 2018 [19]	●	●	●	●	○	●	●	●	●	●
Sims et al., 2013 [23]	●	●	●	◐	◐	●	●	●	●	●
Skov-Ettrup et al., 2014 [16]	●	●	●	○	○	●	●	●	●	●
Travis & Lawrance, 2009 [27]	●	●	●	○	○	●	●	●	●	●
Tuisku et al., 2016 [28]	●	●	●	●	◐	●	●	●	●	●
Vogel et al., 2019 [20]	●	●	●	N/A	N/A	●	●	●	●	●
Ybarra et al., 2013 [17]	●	●	●	●	◐	●	●	●	●	●
Zanis et al., 2011 [24]	●	●	●	●	○	●	●	◐	●	●

Note. ● = Elements are completely addressed; ◐ = Elements are partially addressed; ○ = Elements are not addressed. N/A: Not applicable

Note: Elements for evaluation in each domain are as follows: ^a^Study question: Is the study question clearly focused and appropriate? ^b^Study population: Is the study population well described? Are inclusion and exclusion criteria clear? ^c^Comparability of subjects: Is group comparability assured? ^d^Randomization: Is random sequence generation process described? Is adequate concealment method used for randomization? ^e^Blinding: Is double-blinding used to treatment allocation? ^f^ Interventions: Is intervention clearly detailed for all study groups? ^g^Outcomes: Is there primary and secondary outcome measures specified? Is the method of measurement used standard, valid, and reliable? ^h^Statistical analysis: Is there an appropriate analytic technique used to address study withdrawals, loss to follow-up, missing data, and intention to treat? ^I^Results: Are the outcome effect and measures of precision provided? ^j^Discussions: Are conclusions supported by results with the consideration of potential biases of the studies?

## Results

### Search results

From the initial search, 2132 articles were identified. Among them, 542 duplicated articles were removed. Next, the remaining 1590 articles were imported for title and abstract review. Two independent researchers reviewed the title and abstract of each article to assess eligibility. Discrepancies were discussed with between the reviewers, and the first author made a final decision on inclusion. In this process, 1473 irrelevant studies were eliminated, and 117 articles were fully reviewed. Finally, 14 articles meeting the criteria were included in this review (see Fig. 1).

### Overview of the studies

Table 1 summarizes the study settings and sample characteristics. Table 2 describes the study design and control group conditions. The studies were generally based in North America including 7 studies from U.S. and two study from Canada (9/14, 64.3%). The rest of the studies were from Europe (Denmark, Switzerland, Spain, and Finland; 5/14) or Asia (1/14). The sample sizes ranged from 40 to 2030. Age ranged from 18 to 26 years. The proportion of females ranged from 35 to 62%. Most studies used social media (text-based or web-based App) interventions for participants recruited online [18, 22]. Other studies used diverse settings, including universities [25, 27], and hospitals for pharmacological intervention [28]. One study specifically included non-college young adults in rural areas [24]. All but two of the studies used a two-arm randomized controlled design. Three studies compared the intervention group to an assessment-only control group without intervention, and the rest of the studies used attention-matched control interventions or different kinds of interventions in the control group.

### Description of interventions

#### Modes of interventions

 Interventions included the following: text messaging (*n* = 3), social media (*n* = 2), phone or virtual (*n* = 1), or in-person counseling (*n* = 3); pharmacological agent (*n* = 1), social branding (*n* = 3), and a self-help booklet with a booster phone call (*n* = 1). All social media interventions used Facebook as an intervention platform.

#### Theoretical framework

Diverse theoretical frameworks were used to guide each intervention. The transtheoretical model was most commonly used, particularly for social media–based intervention studies [19]. In addition, social cognitive theory was used in one study utilizing SMS to quit tobacco among young adults in United States [18]. Ottawa decision support framework and self-affirmation theory were the theories implemented in the App interventions [21, 22]. Health behavior theories, including the Health Action Process Approach (HAPA) health behavior model, and social cognitive theory and theory of planned behavior formed the basis for text-based interventions [15, 16].

#### Frequency, duration, and intensity

The intervention duration and frequency of contact with participants were variable and yielded varied outcomes (Supplement 1). On average, the duration of the intervention was over 3 months, although shorter durations were used including a single session intervention of direct counseling. For text-based interventions, the duration of the intervention ranged from 6 weeks to 3 months, and the frequency of the intervention varied. One study provided a single text message weekly [15], while in two other studies, the frequency of the messages changed depending on the stage of quitting with the frequency of the text messages gradually increasing until the quit dates and then decreasing over the rest of the intervention interval. [16–18]. For example, one study used four messages per day before the quit date and eight messages per day on the quit date [17]. For social media interventions, messages were provided in the form of one post per day for 3 months [20], and one study provided one smoking-cessation counseling session per week [19]. For phone or virtual counseling, 4 to 10 calls were provided over intervals ranging from 4 weeks to 3 months [23]. The length and frequency of the two studies which relied on in-person interventions differed: one 5-min session versus four sessions of 20–30 min over 4 weeks [24, 25]. Another study combining a booklet and booster phone call had a total duration of 3 months [27]. A pharmacological intervention study was conducted with light and heavy smokers for 8 to 12 weeks using nicotine patches [28]. For App-delivered interventions (mobile or web-based), the interventions were conducted over 6 months: the mobile app used tailored messages with on-demand push notifications based on individual quit plan [21], and the web-based app used questionnaires at baseline, with reassessments at 1- and 6-months [22].

#### Study outcomes

##### Overall quit rates

The quit rates reported among the included studies varied by the outcome definition and the follow-up interval. Most studies relied on self-reported 7-day abstinence rates. Table 2 presents the detailed information. Only 36% of studies reported statistically significant results in terms of quit rates.

For text-based interventions, all four studies reported no significant differences between intervention and control arms in quit rates, except the Graham’s study [15–18]. One study reported quit rates of 44% at 1 month [17], and abstinent rates were 40% based on self-reported 7-day abstinence rates at 3-month follow-up [17]. Another study reported quit rates of 12.5% at 6 months (self-reported 7-day abstinence) and 6.3% (30-day abstinence) [15]. Another study with tailored text messages reported quit rates of 18.8% at 12-month follow-up based on self-reported a 30-day abstinence [16]. In Graham and the colleagues’ study, with intention-to-treat, the 7-month 30-day point prevalence abstinence rate was 24.1% (314 of 1304) among “This is Quit- text message program” participants and 18.6% (239 of 1284) in the control group (Odds ratio = 1.39 (1.15–1.68), *p* < 0.001) [18].

For the social media–based interventions, all two studies reported quit rates based on 7-day abstinence rates. All studies conducted a Facebook-based intervention and compared it to quit rates of the control group referred to the smokefree.gov site. One group tested the intervention with a RCT study design, and quit rates were 13.6%, 18.6%, and 21.8% at 3 months, 6 months, and 1 year based on the self-reported 7-day abstinence [19]. The other study stated a self-reported 7-day abstinence rate of 8.6% for sexual and gender minority (SGM) individuals and 15.4% for non-SGM young adults at 3-month follow-up, and 18.8% and 15.4% at 6-month follow-up, respectively [20]. None of these results were significant.

One study tested a phone or in-person counseling intervention compared to the control group. One RCT study tested the quit line, and yielded biochemically verified quit rates of 11%, 8.1%, and 6.7% at 1-, 3-, and 6-month follow-up (Sims et al., 2013), which were higher than the control group with a self-help booklet–based treatment group.

Two studies used app-based or web-based interventions. In one study, the intervention group used a comprehensive evidence-based smoking cessation smartphone app where participants customized a cessation plan and were reminded of how much money they had saved (Baskereville et al., 2018). Participants in the control group received a self-help guide developed by Health Canada for young adult smokers called Pathways to Quit. Intention-to-treat analysis showed that continuous abstinence (*N* = 1599) assessed at 6 months and 30-day point prevalence abstinence at 6 months were not significantly different (Baskereville et al., 2018). Overall satisfaction, perceived helpfulness, and frequency of use were higher and statistically significant in the control group compared to the intervention group (Baskereville et al., 2018). Another RCT asked participants in the intervention arm to visit the U@Uni website and view online resources on health behaviors in the form of text and video and downloaded the U@Uni smartphone app at the beginning of the second semester while the control group received no resources. The intervention had a statistically significant effect on smoking status (current smoker) at the 6-month follow-up, with fewer self-reported smokers in the intervention group (8.7%) than in the control group (13.0%; OR = 1.92, *p* = 0. 010) [22].

Three studies tested in-person counseling interventions. Zanis and colleagues study tested in-person counseling (brief direct treatment intervention) in comparison to the existing quit line intervention as a control group. Brief direct counseling resulted in 19.8% quit rates in the intervention group, compared to quit rates of 10.2% in the control group. Although the results were not statistically different between the intervention and control groups, only 1.7% of those who were assigned to the control group contacted the quit line for assistance [24]. This study reported a biochemically confirmed 30-day abstinence rate of 19.8% at 3 months in the intervention arm compared to 10.2% in the control arm (*p* > 0.05). Harris et al. (2010) described a self-reported quit rate of 31.4% at post-intervention and a bio-chemically verified 30-day abstinence rate of 20.4% at 6 months, which was not significantly different from the control arm (quit rates of 28.0% and 20.4%, respectively) [25]. Orsal and Ergun examined the effects of peer education on the decision to quit smoking, smoking cessation, self-efficacy, and behavior change among students who applied to the Youth Friendly Center to quit smoking [26]. Results showed that participants in the experimental group had a higher reduction of nicotine addiction, which was statistically significant, and that 94% of students in the intervention group remained free of nicotine addiction and had successfully quit at the seventh and eighth follow-up at time1 and time 2, respectively (*p* < 0.001).

One study provided a booklet-based intervention with a booster phone call with three arms using a RCT [27], and the authors reported a 11.4% self-reported 7-day abstinence rate at 3-month follow-up, compared to the usual care group (2.9%) or a different booklet-based program (5.6%; insignificant). A RCT based on pharmacologic intervention reported a self-reported 7-day abstinence rate of 19.6% among nicotine patch users compared to varenicline (15.7%) [28].

#### Quit attempts and other outcomes

In addition to the overall quit rates, other self-reported outcomes (i.e., quit attempts, readiness to quit, and stage of change) were used to evaluate the potential effects of smoking-cessation interventions with regard to more proximate measures which might support eventual cessation [15, 19, 25]. For example, American college students who received up to four one-on-one MI sessions for smoking cessation reported significantly greater quit attempts at the end of treatment (Odds Ratio = 1.75, 95% CI [1.11, 2.74], *p* = 0.02) and 6-month follow-up (Odds Ratio = 1.66, 95% CI [1.11, 2.47], *p* = 0.01) than college students who received MI for fruits and vegetables [25]. However, such an increase was not seen in the web- or text message–based interventions. For example, the Tobacco Status Project (TSP) Facebook smoking-cessation intervention did not increase quit attempts (Odds Ratio = 0.94, 95% CI [0.22 – 3.73], *p* = 0.929) or the readiness to quit smoking (Odds Ratio = 0.927, 95% CI [0.089 – 9.68], *p* = 0.947) over a period of 12 months among young adults in the United States [19]. In Switzerland, there were no differences in quit attempts (Odds Ratio = 1.18, 95% CI [0.81 – 1.72], *p* = 0.38) and stage of change (*p* = 0.69), whether vocational school students were daily smokers and had received a weekly SMS text-message intervention or not [15].

#### Evaluation of the study qualities

All included articles were examined for quality using the criteria based on the AHRQ quality evaluation guidelines [14]. Details are presented in the Table 3.

The randomization levels were diverse, including individual and school levels, depending on the risk of contamination. There were methodological issues in the randomization and blinding. Most studies did not fully describe the randomization process, including the random sequence–generation process or concealment method used for randomization. In terms of blinding, it was difficult to double blind because of the nature of interventions and controls used in the studies. When various modes or contents were provided, it was almost impossible to blind participants and interventionists to which intervention group they are in.

In terms of intervention, all studies clearly provided detailed description of the study groups, and an appropriate analytic technique was used. One study did not report the outcomes based on intent-to-treat analysis [24]. Outcomes were reported using self-report data or biochemically confirmed. All studies with quit rates at least reported the outcomes for the 3-month follow-up outcome. About half of the studies did not provide bio-chemical verification. One study verified the smoking-cessation outcome by double-checking with the participants’ significant others [17].

## Discussion

Although a variety of smoking-cessation programs for tobacco products exist for young adults including text messaging, social media, app or web-based, phone call or in-person-based counseling, pharmacological, and multicomponent-based, there is a limited evidence base supporting each approach based on a review of the published literature. Moreover, due to study heterogeneity, it is difficult to make comparisons or to conclude which type of intervention(s) is/are most effective for the population of young adult cigarette smokers. Most studies failed to show statistical differences between study arms, perhaps because they were underpowered or perhaps because of a lack of effect. Social media is known as an engaging medium for behavioral interventions among young adults. It is interesting that only limited published studies have used social media–based interventions focused on young adults and that all social media interventions used Facebook as a platform. Given that new types of social media are available, it may be worthwhile to develop an intervention for other platforms. Only two studies used mobile applications even though the young adult population is engaged in technology and digital media.

Another important note is that although this review included only RCTs, the studies relied on variable interventions (e.g., by type, intensity and duration). This review identified only a single RCT examining cessation among e-cigarette users. Given that e-cigarette use is an emerging issue and represents the most prevalent tobacco products among young adults, there are needs to conduct more studies to develop evidence-based interventions for young adults.

In addition, although there is heterogeneity in the overall intervention strategy, intervention conditions (e.g., frequency, duration, and intensity), and outcome measures, there is less variety in the control group condition for each mode of intervention. For example, for social media intervention, the control groups of all three studies were the condition on their referral to smokefree.gov. It is necessary to test the intervention with a diverse, comparable condition to the intervention in order to advance the strength of the evidence. For other types of interventions, only one to three studies were conducted using a randomized design, which limits the production of sufficient evidence for each type of intervention.

Only two studies specifically tested the intervention in subgroups of young adults, including one for those in sexual and gender minority groups, who demonstrate increased rates of smoking [20], and another focused on young adults who are non-college students, as well as those who with lower SES [24]. The tailored intervention for the SGM group, centered on the transtheoretical model, was provided using social media–based intervention, and this study reported a higher abstinence rate among these groups compared to the non-SGM group. In terms of those who are low SES, brief counseling was provided and showed positive results compared to the telephone counseling although the results of these studies were not significant. Considering that each of these subgroups have higher smoking rates, it is necessary to develop further interventions with a tailored approach.

Some methodological gaps have been identified. For instance, a more detailed description of the randomization process, such as sequence generation and concealment, would be beneficial for further studies. In addition, because of the study’s nature (e.g., difference between the intervention and control groups), it is difficult to maintain complete blinding during the RCTs; however, it may be important to develop a method for blinding among the evaluator and participants. In terms of outcome measures, most studies reported a 7-day abstinence rate, which is a standard measurement in smoking-cessation studies. However, it is also recommended to provide a prolonged abstinence rate in order to develop a better picture of the study outcome [29]. In addition, many of the studies relied on self-reported abstinence without bio-verification.

This study has noteworthy limitations. We have used various search terms, including several MeSH terms, to include broader ranges of articles. Keywords choices may have resulted in missing relevant articles. Even though two independent researchers screened for eligibility and the third researcher double-checked the discarded articles, there is a chance of relevant articles have not been included. In addition, there is a chance of error or missing information in the coding process. One researcher initially coded information to provide the raw data, and the other two researchers reviewed the accuracy, which provided a chance to double check information in an effective way. However, this approach may not be the best practice, and the accuracy may be improved by multiple researchers independently performing the coding in the initial process. In addition, because this review was focused on the information provided in the articles, there is a chance that the studies have been misrepresented. For example, studies could have followed all of the appropriate randomization procedures while also struggling to report details of their process due to the journal’s limited space. Moreover, it should be noted that four studies did not specifically provide age ranges. Instead, the studies only stated that they had included university students as participants and they provided the mean ages of the participants. Further, the measures of study outcomes, intervention characteristics (e.g. control group, intervention frequency, and duration), and follow-up periods varied, making it difficult to compare the study outcomes across the studies.

This study provides recommendations for future research and practice. Future research must be conducted to develop more definitive conclusions and stronger evidence for smoking-cessation interventions among young adults. There are only a few studies for each mode of intervention, many did not use randomization and control groups, and a very limited studies have reported statistically significant results. It is important to conduct additional research consisting of a large sample with more rigorous study methods. For example, one study tested pharmacological intervention by comparing varenicline to the nicotine patch, and it produced statistically significant higher quit rates at 1- and 3-month follow-up, particularly for heavy smokers but had significant methodological limitations (e.g., the lack of double blinding and biochemical verification of the outcome measure). The follow-up studies would provide implications for practice changes.

## Conclusion

This systematic review provides a summary of smoking-cessation interventions targeting young adults. Due to the heterogeneous type of interventions, the condition of the intervention and control groups, varied outcome measures, and the small number of publications for each type of intervention, it is difficult to determine the best evidence for smoking cessation for young adults. However, all studies provided positive results in smoking-cessation outcomes compared to the control groups in spite of the lack of statistical significance, which shows the definite need for further research. This review highlights the methodological issues apparent in these studies, such as the measures of study outcomes and the insufficient description of the randomization process. Relatedly, future studies of young adults will need to address the potential use of multiple forms of nicotine delivery (e.g., combustible and non-combustible) which have become popular in this age group. In addition, this study supports the need for further research to identify effective interventions for young adult smokers, including those of lower socioeconomic status, racial/ethnic minority groups, and LGBTQ groups, who generally have higher rates of smoking.

## Supplementary Information

**Additional file 1: Supplement Table 1.** Intervention Characteristics (Frequency, Duration, and Intensity).

## Data Availability

The datasets used and/or analyzed during the current study available from the corresponding author on reasonable request.

## References

[1] Creamer M. R, Wang T. W, Babb S, Cullen K. A, Day H, Willis G, Jamal A, Neff L (2019). Tobacco Product Use and Cessation Indicators Among Adults — United States, 2018. MMWR Morb Mortal Wkly Rep.

[2] U.S. Department of Health and Human Services. (2014). The health consequences of smoking--50 years of progress : a report of the Surgeon General. Department of Health and Human Services, Centers for Disease Control and Prevention, National Center for Chronic Disease Prevention and Health Promotion, Office on Smoking and Health. https://aahb.org/Resources/Pictures/Meetings/2014-Charleston/PPT%20Presentations/Sunday%20Welcome/Abrams.AAHB.3.13.v1.o.pdf.

[3] Doll R, Peto R, Boreham J, Sutherland I. Mortality in relation to smoking: 50 years’ observations on male British doctors. BMJ. 2004;328(7455):1519–28. 10.1136/bmj.38142.554479.AE. 10.1136/bmj.38142.554479.AE PMC437139 15213107

[4] Centers for Disease Control and Prevention. (2020, December 16). Youth and Tobacco Use. https://www.cdc.gov/tobacco/data_statistics/fact_sheets/youth_data/tobacco_use/index.htm.

[5] Pasquereau A, Guignard R, Andler R, Nguyen-Thanh V (2017). Electronic cigarettes, quit attempts and smoking cessation: A 6-month follow-up: Electronic cigarettes, quit attempts and smoking cessation. Addiction (Abingdon, England).

[6] Thrul J, Ramo DE (2017). Cessation Strategies Young Adult Smokers Use After Participating in a Facebook Intervention. Subst Use Misuse.

[7] Watkins SL, Thrul J, Max W, Ling PM (2020). Cold Turkey and Hot Vapes? A National Study of Young Adult Cigarette Cessation Strategies. Nicotine Tob Res.

[8] Institute of Medicine; National Research Council; Bonnie RJ, Stroud C, Breiner H, editors. Washington (DC): National Academies Press (US); 2015 Jan 27. 25855847

[9] Arnett JJ (2004). Emerging adulthood: The winding road from the late teens through the twenties.

[10] Fanshawe T, Halliwell W, Lindson N, Aveyard P, Livingstone‐Banks J, Hartmann‐Boyce J, Fanshawe T (2017). Tobacco cessation interventions for young people. Cochrane Libr.

[11] Villanti A. C, West J. C, Klemperer E. M, Graham A. L, Mays D, Mermelstein R. J, Higgins S. T (2020). Smoking-Cessation Interventions for U.S. Young Adults: Updated Systematic Review. Am J Prev Med.

[12] Moher D, Liberati A, Tetzlaff J, Altman D (2009). Preferred reporting items for systematic reviews and meta-analyses: the PRISMA statement. BMJ.

[13] Covidence (2023). Retrieved from www.covidence.org

[14] West, S., King, V., Carey, T. S., Lohr, K. N., McKoy, N., Sutton, S. F., & Lux, L. (2002). Systems to rate the strength of scientific evidence. Evidence report/technology assessment (Summary)(47), 1–11. PMC4781591 11979732

[15] Haug S, Venzin M. P, Venzin V, Meyer C, John U (2013). Efficacy of a text message-based smoking cessation intervention for young people: A cluster randomized controlled trial. J Med Internet Res.

[16] Skov-Ettrup LS, Ringgaard LW, Dalum P, Flensborg-Madsen T, Thygesen LC, Tolstrup JS (2014). Comparing tailored and untailored text messages for smoking cessation: A randomized controlled trial among adolescent and young adult smokers. Health Educ Res.

[17] Ybarra ML, Holtrop JS, Prescott TL, Rahbar MH, Strong D (2013). Pilot RCT results of stop my smoking USA: A text messaging-based smoking cessation program for young adults. Nicotine Tob Res.

[18] Graham AL, Amato MS, Cha S, Jacobs MA, Bottcher MM, Papandonatos GD (2021). Effectiveness of a Vaping Cessation Text Message Program Among Young Adult e-Cigarette Users: A Randomized Clinical Trial. JAMA Intern Med.

[19] Ramo DE, Thrul J, Delucchi KL, Hall S, Ling PM, Belohlavek A, Prochaska JJ (2018). A randomized controlled evaluation of the tobacco status project, a Facebook intervention for young adults. Addiction (Abingdon, England).

[20] Vogel EA, Thrul J, Humfleet GL, Delucchi KL, Ramo DE (2019). Smoking cessation intervention trial outcomes for sexual and gender minority young adults. Health Psychol.

[21] Baskerville NB, Struik LL, Guindon GE, Norman CD, Whittaker R, Burns C, Hammond D, Dash D, Brown KS (2018). Effect of a Mobile Phone Intervention on Quitting Smoking in a Young Adult Population of Smokers: Randomized Controlled Trial. JMIR Mhealth Uhealth.

[22] Epton T, Norman P, Dadzie A-S, Harris PR, Webb TL, Sheeran P, Julious SA, Ciravegna F, Brennan A, Meier PS, Naughton D, Petroczi A, Kruger J, Shah I (2014). A theory-based online health behaviour intervention for new university students (U@Uni): results from a randomised controlled trial. BMC Public Health.

[23] Sims TH, McAfee T, Fraser DL, Baker TB, Fiore MC, Smith SS (2013). Quitline cessation counseling for young adult smokers: A randomized clinical trial. Nicotine Tob Res.

[24] Zanis DA, Hollm RE, Derr D, Ibrahim JK, Collins BN, Coviello D, Melochick JR (2011). Comparing intervention strategies among rural, low SES, young adult tobacco users. Am J Health Behav.

[25] Harris KJ, Catley D, Good GE, Cronk NJ, Harrar S, Williams KB (2010). Motivational interviewing for smoking cessation in college students: A group randomized controlled trial. Prev Med.

[26] Orsal O, Ergun A (2021). The Effect of Peer Education on Decision-Making, Smoking-Promoting Factors, Self-Efficacy, Addiction, and Behavior Change in the Process of Quitting Smoking of Young People. Risk Management and Healthcare Policy.

[27] Travis HE, Lawrance K-AG (2009). Randomized Controlled Trial Examining the Effectiveness of a Tailored Self-Help Smoking-Cessation Intervention for Postsecondary Smokers. J Am Coll Health.

[28] Tuisku A, Salmela M, Nieminen P, Toljamo T (2016). Varenicline and Nicotine Patch Therapies in Young Adults Motivated to Quit Smoking: A Randomized, Placebo-controlled, Prospective Study. Basic Clin Pharmacol Toxicol.

[29] Hughes JR, Keely JP, Niaura RS, Ossip-Klein DJ, Richmond RL, Swan GE (2003). Measures of abstinence in clinical trials: Issues and recommendations. Nicotine Tob Res.

