# Supplementary material for: Systematic review: interventions to quit tobacco products for young adults
Source: BMC Public Health. 2023 Jun 26;23:1233. doi: 10.1186/s12889-023-15900-8 (PMC10294369; doi:10.1186/s12889-023-15900-8)
Supplement: Supplementary file 1 — Additional file 1: Supplement Table 1. Intervention Characteristics (Frequency, Duration, and Intensity). [file 12889_2023_15900_MOESM1_ESM.docx]

**Supplement Table 1. Intervention Characteristics (Frequency, Duration, and Intensity)**

| Intervention | Studies (Intervention) | Tobacco products | Frequency, Duration, intensity | Control group |
| --- | --- | --- | --- | --- |
| Text-based | Haug et al., 2013 | cigarettes | **3 months** in total  1 message/week | Assessment only control group |
|  | Skov-Ettrup et al., 2014  (Tailored text messages) | cigarettes | **3 months** in total  1^st^ 4 weeks: 1 message/day (1 message/day: 1-3 days before the quit date)  2^nd^ 4 weeks: 2 tailored messages/day  3^rd^ 4 weeks: 4 or 5 messages/week | Untailored text messages  1 message/day over 5 weeks  1 message/week over the following 3 weeks |
|  | Ybarra et al., 2013  (SMS USA) | cigarettes | **6 weeks** in total  Pre-Quit stage:  4 messages/day over 2-week  Early Quit stage:  9 messages /day on Quit day and Post-Quit Day 2  8 messages /day on Post-Quit Day 3  7 messages/day on until the last day of the week  Late Quit stage:  2 messages/day over 2 weeks  1 message/day during the final week | Attention-matched control group (sleep and physical activity) |
|  | Graham et al., 2021  (SMS USA) | vape | Who not ready to quit: receive 4 weeks of messages focused on building skills and confidence  who set a quit date: receive messages for 1 week and 8 weeks afterward | Assessment only, referral to after 7-month follow-up assessment |
| Social media | Ramo et al., 2018  (Tobacco Status Project Facebook) | cigarettes | 1 post/day over **3 months**  1 smoking cessation counseling/week over **3 months**  1, 45-minute cognitive-behavioral treatment session/2 weeks over **3 months** | Referral to Smokefree.gov |
|  | Vogel et al., 2019 | cigarettes | 1 post/day over **3 months** | Referral to Smokefree.gov |
| Mobile app | Baskerville et al., 2018 | cigarettes | **6 months** total  Based on tailored messages based on individual quit plan with on-demand push notifications | an evidence-informed self-help guide, On the Road to Quitting (OnRQ) |
| Web based & mobile app | Epton et al., 2014 | cigarettes | **6 months** total: Questionnaire upon starting University, again at one month, and at 6 months | No intervention |
| Phone or virtual counseling | Sims et al., 2013 (Quit line) | cigarettes | 4 calls over a **4–6- week**  Self-help booklet | Self-help booklet |
| In-person counseling | Zanis et al., 2011  (Brief direct treatment intervention) | cigarettes | 1 brief direct tobacco intervention session  About 5 minutes per session | Telephone Quitline (TQ) |
|  | Harris et al., 2010  (Motivational interviewing with a trained counselor) | cigarettes | 4 one-on-one sessions  20-30 minutes/session  Over **4 weeks** | Attention-matched control group (fruits and vegetables) |
| In-person counseling | Orsal & Ergun, 2021 | cigarettes | Consultation with peer educators every 20 days for **6 months** total | No intervention |
| Booklet-based+ a booster phone call | Travis & Lawrance, 2009 (Smok; Quit) | cigarettes | Over **3 months** | usual care quit kit |
| Pharmacological intervention | Tuisku et al., 2016  (Nicotine patch) | cigarettes | Light smokers: placebo patch for **2 months** (group 1) or nicotine patch 10 mg/16 hr for **2 months** (group 2)  Heavy smokers: stronger nicotine patches 15 mg/16 hr for **2 months** (group 3) or **3 months** of varenicline treatment (group 4) | Placebo patch or nicotine patch |
